# The use of concept maps during knowledge elicitation in ontology development processes – the nutrigenomics use case

**DOI:** 10.1186/1471-2105-7-267

**Published:** 2006-05-25

**Authors:** Alexander Garcia Castro, Philippe Rocca-Serra, Robert Stevens, Chris Taylor, Karim Nashar, Mark A Ragan, Susanna-Assunta Sansone

**Affiliations:** 1 Microarray Informatics Team, The European Bioinformatics Institute – European Molecular Biology Laboratory Outstation, Wellcome Trust Genome Campus CB10 1SD, Cambridge Hinxton, UK; 2 Australian Research Council Centre in Bioinformatics, Institute for Molecular Bioscience, The University of Queensland 4072, St Lucia, Australia; 3 Institute for Molecular Bioscience, The University of Queensland 4072, Brisbane, Australia; 4 Australian Centre for Plant Functional Genomics, The University of Queensland 4072, Brisbane, Australia; 5 School of Computer Science, University of Manchester, Kilburn Building, Oxford Road Manchester M13 9PL, Manchester, UK

## Abstract

**Background:**

Incorporation of ontologies into annotations has enabled 'semantic integration' of complex data, making explicit the knowledge within a certain field. One of the major bottlenecks in developing bio-ontologies is the lack of a unified methodology. Different methodologies have been proposed for different scenarios, but there is no agreed-upon standard methodology for building ontologies. The involvement of geographically distributed domain experts, the need for domain experts to lead the design process, the application of the ontologies and the life cycles of bio-ontologies are amongst the features not considered by previously proposed methodologies.

**Results:**

Here, we present a methodology for developing ontologies within the biological domain. We describe our scenario, competency questions, results and milestones for each methodological stage. We introduce the use of concept maps during knowledge acquisition phases as a feasible transition between domain expert and knowledge engineer.

**Conclusion:**

The contributions of this paper are the thorough description of the steps we suggest when building an ontology, example use of concept maps, consideration of applicability to the development of lower-level ontologies and application to decentralised environments. We have found that within our scenario conceptual maps played an important role in the development process.

## Background

In the field of biological research, recent advances in functional genomics technologies have given the opportunity to carry out complex and possibly high-throughput investigations. Consequently, the storage, management, exchange and description of data in this domain present challenges to biologists and bioinformaticians. It is widely recognized that capturing descriptions of investigations at a high level of granularity is necessary to enable efficient data sharing and meaningful data mining [1,2]. However, this information is often captured in diverse formats, mostly as free text, and is commonly subject to typographical errors. The increased cost of interpreting the experimental procedures and exploring data has encouraged several scientific communities to develop and adopt ontology-based knowledge representations to extend power of their computational approaches [3].

Application of an ontologically based approach should be more powerful than simple keyword-based methods for information retrieval. Not only can semantic queries be formed, but axioms that specify relations among concepts can also be provided, making it possible for a user to derive information that has been specified only implicitly. In this way, relevant entries and text can be found even if none of the query words is present (*e.g*. a query for "furry quadrupeds" might retrieve pages about bears) [4].

Many methodologies for building ontologies have been described [5] and seminal work in the field of anatomy provides insights into how to build a successful ontology [6,7]. Extensive work about the nature of the relations that can be used also provides solid grounds for consistent development for building ontologies [8]. However, despite these efforts, bio-ontologies still tend to be built on an *ad hoc *basis rather than by following a well-defined engineering process. To this day, no standard methodology for building ontologies has been agreed upon. Usually terminology is gathered and organised into a taxonomy, from which key concepts are identified and related to create a concrete ontology. Case studies have been described for the development of ontologies in diverse domains, although surprisingly only one of these has been reported to have been applied in a domain allied to bioscience – the chemical ontology [9] – and none in bioscience *per se*. Most of the literature focuses on issues such as the suitability of particular tools and languages for building ontologies, with little attention being given to *how *it should be done. This is almost certainly because the main interest has been in reporting content and use, rather than engineering methodology. Nevertheless, it is apparent that most ontologies are built with the ontological equivalent of "hacking".

A particular lack in these methodologies is support for the continued involvement of domain experts scattered around the world. Biological sciences pose a scenario in which domain experts are geographically distributed, the structure of the ontology is constantly evolving, and the role of the knowledge engineer is not that of the leader but more of the one who promotes collaboration and communication among domain experts. Bioinformatics has demonstrated a need for bio-ontologies and several characteristics highlight the lack of support for these requirements:

• the volatility of knowledge in the domain – biologists' understanding of the domain is in continual flux;

• the domain is large, complex, and cannot, therefore be modelled in one single effort; the knowledge holders are distributed and will not be brought together for frequent knowledge elicitation exercises.

To support these requirements, our methodology pays particular attention to the knowledge elicitation stage of the process of building an ontology. This is the stage where the person managing the development of the ontology gathers, in the form of concepts and relationships between concepts, what the domain expert understands to exist in that domain. To do this, we used concept maps (CMs), a simple graphical representation in which instances and classes are presented as nodes, and relationships between them are shown as arcs [10]. CMs have a simple semantics that appears to be an intuitive form by which domain experts can convey their understanding of a domain. We exploit this feature in order to perform the informal modelling stage of building an ontology.

In support of this argument, we first present a survey of ontology development methodologies, and then report our experience, with particular focus on the *how *of the initial stages of building an ontology using CMs. We have studied and evaluated the key methodologies and have adapted parts of several of them to produce an overall method, which we describe here as a set of detailed stages that, we argue, can be applied to other domains within the biological sciences. The major contributions of this paper are the thorough description of our methodology for building an ontology (including an examination of the utility of CMs), the consideration of its applicability to the development of ontologies, and the assessment of its suitability for use in decentralised settings. Finally, we discuss the issues raised and draw conclusions.

### A survey of methodologies

We investigated five methodologies: Enterprise Methodology [11], TOVE (TOronto Virtual Enterprise) [12,13], the Unified Methodology [14,15], Diligent [16] and Methontology [17]. Table 1 presents a summary of our comparison. We analyzed these approaches according to the following criteria:

- Accuracy in the description of the stages: We were interested in knowing if the stages were sufficiently described so they could be easily followed.

- Terminology extraction: We wanted to study how could terminology extraction assist knowledge engineers and domain experts when building ontologies. We were interested in those methodologies that could offer some level of support for identifying terms.

- Generality: We needed to know how dependent on a particular intended use the investigated methodologies are. This point was of our particular interest since our ontology was intended to serve a particular task. This parameter may be assimilated to the ability of the method to be applied to a different scenario, or use of the ontology it self.

- Ontology evaluation: We needed to know how could we evaluate the completeness of our ontology. This point was interesting for us since we were working with agreements within the community, and domain experts could therefore agree upon errors in the models.

- Distributed *and *decentralized: We were interested in those methodologies that could offer support for communities such as ours in which domain experts were not only geographically distributed but also organized in an atypical manner (*i.e*. not fully hierarchical).

- Usability: We had a particular interest in those methodologies for which real examples had been reported. Had the methodology been applied to building a real ontology?

- Supporting software: We were interested in knowing whether the methodology was independent from particular software.

We found that only Diligent offered community support for building ontologies and none of them had detailed descriptions about knowledge elicitation, nor did they have details on the different steps that had to be undertaken. The methodologies mentioned above have been applied mostly in controlled environments where the ontology is deployed on a one-off basis. Tools, languages and methodologies for building ontologies has been the main research goal for many computer scientists; whereas for the bioinformatics community, it is just one step in the process of developing software to support tasks such as annotation and text mining Unfortunately, none of the methodologies investigated was designed for the requirements of bioinformatics, nor has any of them been standardised and stabilised long enough to have a significant user community (i.e. large enough for the ontology to have an impact on the community) [18]. Theoretically, the methodologies are independent from the domain and intended use. However, none of the methodologies has been used long enough as to provide evidence of its generality. They had been developed in order to address a specific problem or as an end by it self. The evaluation of the ontology remains a difficult issue to address; there is a lack of criteria for evaluating ontologies. Within our particular scenario, the models were being built upon agreements between domain experts. Evaluation was therefore based upon their knowledge and thus could contain "settled" errors. We studied those knowledge elicitation methods described by [19] such as observation, interviews, process tracing, conceptual methods, and card sorting. Unfortunately, none of them was described within the context of ontology development in a decentralised setting.

We drew parallels between the biological domain and the Semantic Web (SW). This is a vision in which the current, largely human-accessible Web, is annotated from ontologies such that the vast content of the Web is available to machine processing [20]. Pinto and coworkers [21] define these scenarios as distributed, loosely controlled and evolving. Domain experts in biological sciences are rarely in one place; they tend to form virtual organizations where experts with different but complementary skills collaborate in building an ontology for a specific purpose. The structure of the collaboration does not necessarily have a central control and different domain experts join and leave the network at any time and decide on the scope of their contribution to the joint effort. Biological ontologies are constantly evolving, not only as new instances are added, but also as new whole/part-of properties are identified as new uses of the ontology are investigated. The rapid evolution of biological ontologies is due in part to the fact that ontology builders are also those who will ultimately use the ontology [22].

Some of the differences between classic proposals from Knowledge Engineering (KE) and the requirements of the SW, have been presented by Pinto and coworkers [21], who summarise these differences in four key points:

1. Distributed information processing with ontologies: within the SW scenario, ontologies are developed by geographically distributed domain experts willing to collaborate, whereas KE deals with centrally-developed ontologies.

2. Domain expert-centric design: within the SW scenario, domain experts guide the effort while the knowledge engineer assists them. There is a clear and dynamic separation between the domain of knowledge and the operational domain. In contrast, traditional KE approaches relegate the role of the expert as an informant to the knowledge engineer.

3. Ontologies are in constant evolution in SW, whereas in KE scenarios, ontologies are simply developed and deployed.

4. Additionally, within the SW scenario, fine-grained guidance should be provided by the knowledge engineer to the domain experts.

We consider these four points to be applicable within biological domains, where domain experts have crafted ontologies, taken care of their evolution, and defined their ultimate use. Our proposed methodology takes into account all the considerations reported by Pinto and coworkers [21], as well as those previously studied by the knowledge representation community.

## Methods

### General view of our methodology

**Figure 1:**
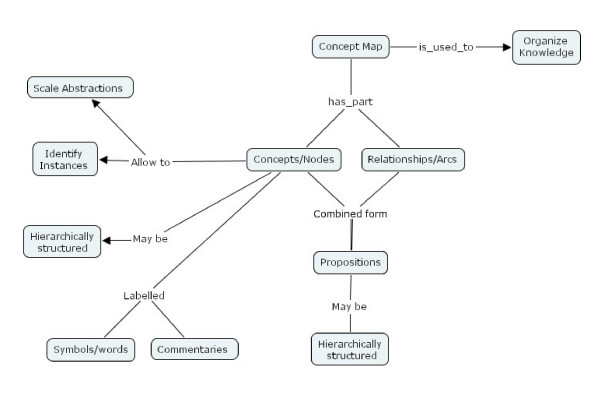
View of a concept map. Adapted with permission from [10].

A key feature of our methodology is the use of CMs throughout our knowledge elicitation process. CMs are graphs consisting of nodes representing concepts, connected by arcs representing the relationships between those nodes [23]. Nodes are labelled with text describing the concept that they represent, and the arcs are labelled (sometimes only implicitly) with a relationship type. CMs proved, within our development, useful both for sharing and capturing activities, and in the formalisation of use cases. Figure 1 illustrates a CM.

**Figure 2:**
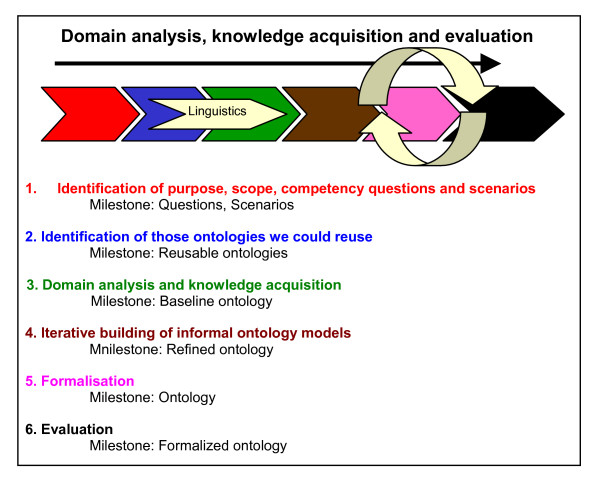
Steps (1–6) and milestones (boxes).

Our methodology strongly emphasises: (*i*)capturing knowledge, (*ii*) sharing knowledge, (*iii*) supporting needs with well-structured use cases, and (*iv*) supporting collaboration in distributed (decentralised) environments. Figure 2 presents those steps and milestones that we envisage to occur during our ontology development process.

Step 1: The first step involves addressing straight forward questions such as: what is the ontology going to be used for? How is the ontology ultimately going to be used by the software implementation? What do we want the ontology to be aware of, and what is the scope of the knowledge we want to have in the ontology?

Step 2: When identifying reusable ontologies, it is important to focus on what any particular concept is used for, how it impacts on and relates to other concepts, how it is embedded within the process to which it is relevant, and how domain experts understand it. It is not important to identify exact linguistic matches. By *recyclability *of different ontologies, we do not imply that we can indicate which other ontology should be used in a particular area or problem; instead, we mean conceptually how and when one can extrapolate from one context to another. Extrapolating from one context to another largely depends on the agreement of the community, and specific conditions of the contexts involved. Indicating where another ontology should be used to harmonise the representation at hand – for example, between geographical ontologies and the NCBI (National Center for Biotechnology Information) taxonomy – is a different issue that we refer to as *reusability*.

Step 3: Domain analysis and knowledge acquisition are processes by which the information used in a particular domain is identified, captured and organised for the purpose of making it available in an ontology. This step may be seen as the 'art of questioning', since ultimately all relevant knowledge is either directly or indirectly in the heads of domain experts. This step involves the definition of the terminology, *i.e*. the linguistic phase. This starts by the identification of those reusable ontologies and terminates with the baseline ontology, *i.e*. a draft version containing few but seminal elements of an ontology. We found it important to maintain the following criteria during knowledge acquisition:

• *Accuracy *in the definition of terms. The linguistic part of our development was also meant to support the sharing of information/knowledge. Table 2 presents the structure of our linguistic definitions. The availability of context as part of the definition proved to be useful when sharing knowledge.

• *Coherence: *as CMs were being enriched it was important to ensure the coherence of the story we were capturing. Domain experts were asked to use the CMs as a means to tell a story; consistency within the narration was therefore crucial.

• *Extensibility: *Our approach may be seen as an aggregation problem; CMs were constantly gaining information, which was always part of a bigger narration. Extending the conceptual model was not only about adding more details to the existing CMs, nor it was it just about generating new CMs; it was also about grouping concepts into higher-level abstractions and validating these with domain experts. Scaling the models involved the participation of both domain experts and the knowledge engineer. It was mostly done by direct interview and confrontation with the models from different perspectives. The participation of new "fresh" domain experts as well as the intervention of experts from allied domains allowed us to analyse the models from different angles. This participatory process allowed us to re-factorise the models by increasing the level of abstraction.

The goal determines the complexity of the process. Creating an ontology intended only to provide a basic understanding of a domain may require less effort than creating one intended to support formal logical arguments and proofs in a domain. We must answer questions such as: Why are we building this ontology? What do we want to use it for? How is it going to be used by the software layer? Subsections *Identification of purpose, scope, competency questions and scenarios *to *Iterative building of informal ontology models *explain these steps in detail.

Step 4: Iterative building of informal ontology models helped to expand our glossary of terms, relations, their definition or meaning, and additional information such as examples to clarify the meaning where appropriate. Different models were built and validated with the domain experts.

Step 5: Formalisation of the ontology was the step during which the classes were constrained, and instances were attached to their corresponding classes. For example: "a male is constrained to be an animal with a y-chromosome". This step involves the use of an ontology editor.

Step 6: There is no unified framework to evaluate ontologies, and this remains an active field of research. We consider that ontologies should be evaluated according to their fitness for purpose, *i.e*. an ontology developed for annotation purposes should be evaluated by the quality of the annotation and the usability of the annotation software. By the same token, the recall and precision of the data, and the usability of the conceptual query builder, should form the basis of the evaluation of an ontology designed to enable data retrieval.

### Scenarios and ontology development process

The methodology we report herein has been applied during the knowledge elicitation phase with the European nutrigenomics community (NuGO)[24]. Nutrigenomics is the study of the response of a genome to nutrients, using "omics" technologies such as genomic-scale mRNA expression (transcriptomics), cell and tissue-wide protein expression (proteomics), and metabolite profiling (metabolomics) in combination with conventional methods. NuGO includes twenty-two partner organisations from ten European countries, and aims to develop and integrate all facets of resources, thereby making future nutrigenomics research easier. An ontology for nutrigenomics investigations would be one of these resources, designed to provide semantics for those descriptors relevant to the interpretation and analysis of the data. When developing an ontology involving geographically distributed domain experts, as in our case, the domain analysis and knowledge acquisition phases may become a bottleneck due to difficulties in establishing a formal means of communication (*i.e*. in sharing knowledge).

Additionally, the NuGO participants collaborate with international toxicogenomics and environmental genomics communities under the RSBI (Reporting Structure for Biological Investigations) [25], a working group of the Microarray Gene Expression Data (MGED) Society. One of the objectives of RSBI is the development of a common high-level abstraction defining the semantic and syntactic scaffold of a record/document that describes an investigation in these diverse biological domains. The RSBI groups will validate the high-level abstraction against complex uses cases from their domain communities, ultimately contributing to the Functional Genomics Ontology (FuGO), a large international collaborative development project [26].

Application of our methodology in this context, with geographically distributed groups, has allowed us to examine its applicability and understand the suitability of some of the tools currently available for collaborative ontology development.

#### Identification of purpose, scope, competency questions and scenarios

Whilst the high-level framework of the nutrigenomics ontology will be build as a the collaborative effort with the others MGED RSBI groups, the lower-level framework aims to provide semantics for those descriptors specific to the nutritional domain.

Having defined the scope of the ontology we discussed the competency questions with our nutrigenomics researchers (henceforth our domain experts); these were used at a later stage in order to help evaluate our model. Examples of those competency questions are presented in table 3.

Competency questions are understood here as those questions for which we want the ontology to be able to provide support for reasoning and inferring processes. We consider ontologies do not answer questions, although they may provide support for reasoning processes. Domain experts should express the competency questions in natural language without any constraint.

#### Identification of reusable and recyclable ontologies

For our particular purposes, we followed a 'top-down' approach where experts in the biological domain work together to identify key concepts, then postulate and capture an initial high-level ontology. We identified for example the Microarray Gene Expression Data (MGED) Ontology (henceforth, MO) [27] as a possible ontology from which we could recycle – extrapolate from one context to another- some terms and/or structure for investigation employing other omics technologies in addition to expression microarrays. The Open Biomedical Ontologies project (OBO) [28,29] was an invaluable source of information for the identification of possible orthogonal ontologies. Domain experts and the knowledge engineer worked together in this task; in our scenario, it was a process where we focused on those high-level concepts that were part of MO and relevant for the description of a complete investigation. We also studied the structure that MO proposes, and by doing so came to appreciate that some concepts could be linguistically different but in essence mean very similar things. This is an iterative process currently done as part of the FuGO project. FuGO will expand the scope of MO, drawing in large numbers of experimentalists and developers, and will draw upon the domain-specific knowledge of a wide range of biological and technical experts.

#### Domain analysis and knowledge acquisition

We hosted a series of meetings during which the domain experts discussed the terminology and structure used to describe nutrigenomics investigations. For us, domain analysis is an iterative process that must take place at every stage of the development process. We focused our discussions on specific descriptions about what the ontology should support, and sketched the planned area in which the ontology would be applied. Our goal was also to guide the knowledge engineer and involve that person in a more direct manner.

An important outcome from this phase was an initial consensus reached on those terms that could potentially have a meaning for our intended users. The main aim of these informal linguistic models was to build an explanatory dictionary; some basic relations were also established between concepts. We decided to use two separate tools (Protégé [30] and CMAP-tools [10]) because none of the existing Protégé plug-ins provided direct manipulation capabilities over the concepts and the relations among them the way CMAP-tools does. Additionally, we studied different elicitation experiences with CMs such as [31,32]. Our knowledge formalism was Description Logic (DL), we used the Protégé OWL plug-in.

CMs were used in two stages of our process: capturing knowledge, and testing the representation. Initially we started to work with informal CMs; although they are not computationally enabled, for a human they appear to have greater utility than other forms of knowledge representation such as spreadsheets or word processor tables. As the model gained semantic richness, by formalising '*is-a*' and '*whole/part-of*' relationships between the concepts the CMs evolved and became more complex. Using CMs, our domain experts were able to identify and represent concepts, and declare relations among them. We used CMAP-tools version 3.8 [10] as a CM editor.

#### Attributes of the domain experts

Experts should of course be highly knowledgeable in their respective areas. We identified two kinds of nutrigenomics experts: high-level experts, scientists at a project coordination level involved in interdisciplinary efforts, and domain-specific experts, with extensive hands-on experience, experimentalists at a more technical level. When developing an ontology, it is also important to have experts with broad vision, so the flow of information could be captured and specific controlled vocabularies properly identified.

#### The knowledge elicitation sessions

The goal of these sessions was to identify both the high-level and low-level domain concepts, why these concepts were needed, and how they could be related. A secondary goal was to identify reusable ontologies where possible.

In the first sessions, it was important to see clearly the 'what went where', as well as the structure of the relationships that 'glued' the information together. We were basically working with informal artefacts (CMs, word processor documents, spreadsheets and drawings); it was only at a later stage that we achieved some formalisation.

Some sessions took place by teleconference; these were supported by iterative use of WEBEX (web, video, and teleconferencing software) [33] and Protégé. CMs were also used to present structural aspects of the concepts. We found it important to set specific goals for each teleconference, with these goals ideally specified as questions that are distributed prior to the meeting. In our case, most of the teleconferences focused on specific concepts, with questions of the form "*how does A relate to B*?", "*why do we need A here instead of B*?", and "*how does A impact on B?*". Cardinality issues were also discussed.

#### Representing conceptual queries

**Figure 3:**
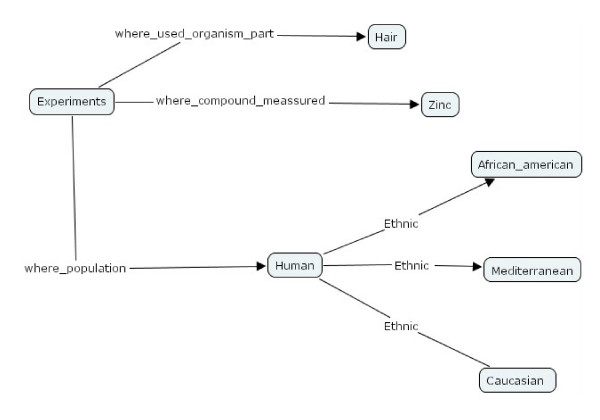
CMs as means to structure a conceptual query.

We also used CMs to represent conceptual queries. We observed that domain experts are used to querying information systems using keywords, rather than building structured queries. In formalising the conceptual queries, CMs provided the domain experts with a tool that allowed them to go from an instance to the appropriate class/concept, at the same time identifying the relationships. For example, within the nutrigenomics domain some investigations study the health status of human volunteers looking at the level of zinc in their hair. These investigations may take place in different research institutes, but all the information may be stored in just one central repository. In order to correlate all those investigations the researcher should be able to formulate a simple query "*what is the zinc concentration in hair across three different ethnic groups*". Figure 3 illustrates this query. Conceptually this query relates *compounds*, *health function *and *ethnicity*. The concept of compound implies a measurement; by the same token the concept of health function implies a particular part of the organism.

Conceptual queries are based on high-level abstractions, relationships between concepts, concept-instances and logical operators; the selection of high-level abstraction allows the class to be instantiated. Conceptual queries provide a level of interaction between the user and the external sources, removing the need for the user to be aware of the schema. We do not want only to guide the user by allowing him/her to select concepts, but would also like to ask the user in a consistent and coherent way so the user can constrain the query before execution takes place, and/or navigate intelligently across terms. Thus, we see why we need an ontology ultimately and not simply a controlled vocabulary, nor merely a dictionary of terms. Controlled vocabularies *per se *describe neither relations among entities nor relations among concepts, and consequently cannot support inference processes [4].

The collected competency questions could be used as a starting point for building the conceptual queries. Competency questions are informal, whereas conceptual queries are used to identify the 'class-relation-instance' and thus improve the understanding of how users may ultimately query the system. Conceptual queries may be understood as a formalisation of competency questions.

#### Iterative building of informal ontology models

**Figure 4:**
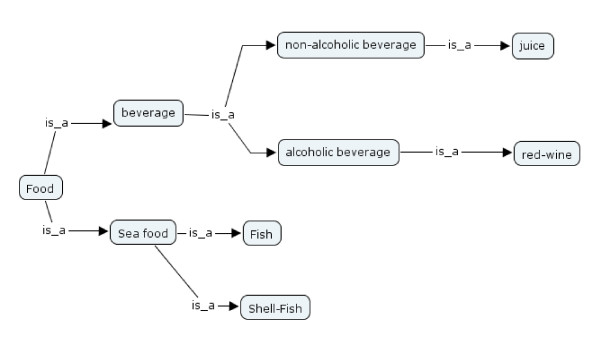
Elicitation of Is_a, whole/part-of, and classes.

Domain experts represented their knowledge in different CMs that they were generating. Their representation was very specific; they were providing instances and relating these instances with very detailed *whole/part-of *relations. Figure 4 presents an example from the nutrigenomics domain that illustrates how we used the CMs in order to move from *instances *to *classes*, to identify *is_a *and defining the *whole/part-of *relationship more precisely.

Initially, domain experts represented specific cases with instances rather than classes. The specificity of the use cases made it easy to identify a *subject-predicate *structure where subjects could be assimilated to instances. Alternatively, predicates in most of the cases had relations and/or information pointing to other ontologies that were needed. Subjects were understood as those entities that perform an action or who receive the action, whereas the predicate contains whatever may be said about the subject.

By gathering use cases in the form of CMs, we could identify the classes and subclasses, for example:*beverage is_a food*, *juice is_a non-alcoholic beverage*. The *has_attribute/is_ attribute_of *property attached to the instance was also discussed. Moving from instances to classes was an iterative process in which domain experts were representing their knowledge by providing a narration full of instances, specific properties, and relationships. The knowledge engineer analysed all the material. By doing so, different levels of abstractions that could be used in order to group those instances were identified; ultimately domain experts validated this analysis.

## Future work

As the nutrigenomics work contributes to the development of FuGO, the final steps -formalisation and evaluation- will be possible only at a later stage, after our results (*e.g. *new concepts and/or structures) are evaluated and integrated into the structure of the functional genomics investigation ontology. However, we will continue to evaluate our framework with our nutrigenomics users and the other RSBI groups, to see if it accurately captures the information we need, and if our terminology and definitions are sufficiently clear to assist the annotation process.

### Formalisation

Moving from informal models to formal models with accurate *is-a *and *whole/part-of *relationships will be done using Protégé. FuGO will also be developed in Protégé because it has a strong community support, multiple visualisation facilities, and it can export the ontology in different formats (*e.g. *OWL, RDF, XML, HTML). Partly because Protégé and CMAP-tools are not currently integrated and partly because they aim to assist different stages during the process of developing an ontology, this has to be done, mostly, by hand. We envisage that integration of these two tools may help knowledge engineers in this process; semi-automated translation from CMs into OWL structures through the provision of assistance, in order to allow developers to formally encode bio-ontologies, would be desirable.

Hayes and coworkers [34] addressed the problem of moving from CMs into OWL models. They extend CMAP-tools so it supports import and export of machine-interpretable knowledge formats such as OWL. Their approach assumes that the construction of the ontology starts from the CM and that the CM evolves naturally into the ontology. This makes it difficult for large ontologies where several CMs shape only a part of the whole ontology. Furthermore, adding asserted conditions (such as *necessary*, *necessary and sufficient*) was not possible; formalisation involves the encoding of the CM into a valid OWL structure by identifying and properly declaring classes and properties. Based on those experiences in which we have used CMs, we are designing a tool that supports such transition.

Difficulties arise from the divergence of syntactic formats between CMs and OWL models; CMs do not have logical constraints, whereas OWL structures are partially supported by them; the lack of connection between concepts as understood in CMs and OWL classes should also be noticed. During the elicitation process, the information gathered by means of CMs was usually incomplete in the sense that it tended to be too narrow -meaningful within the context of a particular researcher. Moreover, CMs were initially picturing processes and at later stages as they were gaining specificity the identification of terms and relationships was being enriched. All of these add to the difference between the information one could gather in a CM and an OWL model. They also emphasises the complementary relationship between one and the other. The *node-arc-node *structure of a CM may be assimilated to an RDF representation as well as to an embryonic OWL model. The proximity between both CMs and OWL models allows the arrangement of a CM directly into the syntactic structure of an OWL file thereby avoiding thus some of the inconveniences of translations between non-related models. The transition from a CM model to an OWL model may be made easier by allowing domain experts to develop parts of the ontology with the assistance of knowledge engineers.

The assistance of the knowledge engineer should focus on the consistency of the whole/part-of properties in order to ensure orthogonality. Domain experts express in their CMs their different views of the world; the fragmentation of the domain of knowledge is mostly done by means of is-a relationship and whole/part-of properties. Once these properties and relationships are properly defined, combining complementary CMs may be much easier; also by doing so, the consistency of the OWL model may be assured.

It will not be only by integrating CM functionality into Protégé that the knowledge acquisition process will be better supported and the formalisation/encoding of ontologies might be achieved more rapidly. It is also important to harmonise both CMs and OWL models syntactically and semantically. The construction of the class hierarchy should be done in parallel with the definition of its properties. This will allow us to identify potential redundancies and inconsistencies in the ontology. Domain analysis will thus be present throughout the whole development process.

### Evaluation

Before putting the ontology into use, we will need to evaluate how accurately it could answer our competency questions and conceptual queries. To accomplish this, we will use CMs as well as some functionalities included in Protégé.

Because our CMs represent the conceptual scaffold of the knowledge we are representing, we will use them to evaluate how this discourse may be mapped into the concepts and relationships we have captured. The rationale behind this is simple: the concepts and relationships, if accurate, may then be mapped into the actual discourse. By doing this we hope to identify:

- Where the concepts are not linguistically clear.

- Whether any redundancies are present.

- Whether the process has been accurately represented both syntactically and semantically.

We envisage a simple structure for our validation sessions: domain experts will be presented with the CM, and asked to map their narration into that CM. Minimal or no help should then be given to the domain expert. The use of CMs as a narrative tool for evaluation of ontologies has not to our knowledge been reported previously. Further research into this particular application of CMs may be valuable.

Ultimately the ontology may also be evaluated by using the PAL (Protégé Axiom Language) plug-in provided by Protégé. PAL allows the construction of more-sophisticated queries. Among those methods described by [35] we checked the consistency using only RACER [36].

## Discussion

Building ontologies is a non-trivial task that depends heavily on domain experts. The methodology presented in this paper may be used in different domains with scenarios similar to ours. We used conceptual maps at different stages during this process, and in different ways. The beauty of CMs is that they are informal artefacts; introducing formal semantics into them remains a matter for further investigation. The translation from CMs to OWL remains manual, and we acknowledge that some information may be lost, or even created, in this step despite the constant participation of domain experts. An ideal ontology development tool would assist users not only during knowledge elicitation, as CMAP-tools does well, but also during the formalisation process, so that everything could be done within one software tool.

On the 'art of questioning': When to ask? How to ask? How to intervene in a discussion without taking sides? These are some of the considerations the elicitor must bear in mind during the sessions. When to ask? Basically he/she should ask only when the discussion is not heading in the direction of answering the stated question. How to ask? The question may be stated as a direct question, or as a hypothesis in the form of, '*if A happens then what happens to B?*', '*what is the relationship between A and B?*', '*what are the implications A may have over B?*'. The knowledge engineer should ideally intervene in discussions as little as possible. The experts are presented with an initial scenario or question, after which their discussion takes place so knowledge can start to be elicited. CMs proved to be a very powerful tool for constraining the discussions in a consistent way.

Unfortunately, too little attention has been paid in the bio-ontological literature to the nature of such relations and of the relata that they join together [8]. This is especially true for ontologies about processes. OBO provides a set of guidelines for structuring the relationships, as well as for building the actual ontology. We are considering these and will follow these guiding principles in our future development. We will also consider the issue of *orthogonality *very carefully, as we have always thought about those ontologies that could, at a later stage, be integrated into our proposed structure.

Currently, knowledge is commonly exchanged *via *email, WIKI pages and teleconferences. Where this may still work for closely related groups or when working within a well-defined domain, we have demonstrated in this paper that CMs could effectively assist both domain experts and the knowledge engineer, and provide a basis for properly visualising the argument and its follow-ups. Tempich and coworkers addressed some of these issues by proposing an argumentation ontology for distributed, loosely-controlled and evolving engineering processes [16,37].

The development of an ontology for Genealogy Management Systems (GMS) was another scenario in which our methodology was applied during the knowledge elicitation process [38]. This was a slightly different scenario because our domain experts were mostly in one place. The GMS ontology is meant to partially support annotation of germoplasm throughout the entire transformation process that takes place in several research institutes. CMs were here initially used in order to represent those different transformation processes, and at a later stage CMs, in combination with semi-automatic terminology extraction algorithms, were also used in order to capture and organise vocabulary. The combination of CMs and these semi-automatic methods for terminology extraction proved to be quite useful; initially domain experts were presented with lists of terms, and were later requested to organise them using CMs.

During the development of the GMS ontology, a *narrative *approach was also investigated in conjunction with semi-automatic text extraction methods. The approach taken was simple: domain experts were asked to build stories as they were providing vocabulary. Empirical evidence from this experience suggests that CMs may provide us with a framework for larger terminology extraction and validation efforts. A paper describing these experiences is in preparation. Despite the differences between those domains, the CMs proved to be useful when capturing and sharing knowledge, both as an external representation of the topic being discussed, and as an organisational method for knowledge elicitation. It should be noticed, however, that only time will tell about the transposability of this methodology into other domains.

## Conclusion

We have focused our efforts on knowledge elicitation within the nutrigenomics community. We present a methodology for building ontologies and report our experiences during the knowledge elicitation phase in particular. An informal evaluation of the knowledge elicitation sessions suggests strong commonalities with the argumentative structure proposed by several authors [21,16,37]. We identify the need for further research on how to manage this arrangement. For instance, it could be desirable to track discussions in a more structured and conceptual manner rather than browsing through a vast set of emails. The structure of discussions over ontologies may follow a pattern. We consider that structuring discussions requires technology to be able to provide some cognitive support to users, not only to post their comments but also to follow and search the threads. Having provided evidence for the applicability of our methodology, it would be interesting to see how it can be extended and better supported by software tools such as Protégé.

Those general-purpose collaborative development environments focus more on technical aspects such as consistency and version control rather than on the actual act of the collaboration. Collaborative environments such as WIKIs or version-control software (*e.g. *configuration management software) do not support ontology development in any special way. Recent developments of Protégé, such as the one proposed by [39] and [19], are an interesting step in the right direction; however too little attention has been placed on the actual process of collaboration when building ontologies within decentralised environments. Diaz and coworkers [39] have developed a tool that provides some extended multi-user capability, sessions, and a versioning control system. Building ontologies in which domain experts are informants and, at the same time, leaders of the process is, however, a more complex process that requires more than just a tool in which different users may edit and work on the same file. Hayes and collaborators. [19] provide an extension to CMAP-tools in which CMs may be saved as an OWL file. However, It proved to be difficult to read these files in Protégé due to some inconsistencies in the generated OWL structure; unfortunately this extension does not provide a way in which it is possible to fully exploit DL. Both Hayes and Diaz, propose interesting solutions. However, we consider collaboration emerges naturally when domain experts are provided with the tools that allow them to represent and share their knowledge in such a way that it is easy to promote and support discussion and concentrate on concepts and constraints. There is a need to support collaborative work from the perspective of allowing users to make use of a virtual working place; cognitive support is therefore needed. The design and development of such a collaborative environment and an accompanying CM plug-in for Protégé that supports both the knowledge acquisition phase and the translation from the CM to an OWL structure are clearly desirable. The development of this plug-in, as well as a more comprehensive collaborative environment, is currently in progress.

Ontologies are constantly evolving, and the conceptual structures should be flexible enough as to allow this dynamic. It is important to report methodological issues (or just "methodology") as part of those papers presenting ontologies, in a section analogous to the "methods and materials" sections required in experimental papers. The added clarity and rigour that such presentation would bring would help the community extend and better adapt existing methodologies, including the one we describe here.

## Authors' contributions

SAS conceived of and coordinated the project. AGC was a knowledge engineer during his 11-month student project at EBI. PRS coordinated the nutrigenomics community within MGED RSBI, and organised and participated in the knowledge elicitation exercises. KN contributed to the knowledge elicitation exercises. RS assisted AGC in conceptualising the methodology, SAS and PRS supervised the knowledge elicitation exercises and, with CT, the associated meetings. AGC wrote the initial version of the manuscript; contributions and critical reviews by the other authors, in particular SAS and RS, delivered the final manuscript.

## Figures and Tables

**Table 1:** Comparison of methodologies.

	**Enterprise Methodology**	**TOVE Methodology**	**Unified Methodology**	**Methontology**	**Diligent**
**Description of stages**	High-level description of stages	Detail is provided for those ontologies developed with this methodology	High-level description of stages	Stages are described for the chemical ontology	High level description
**Terminology extraction**	N/A	N/A	N/A	N/A	N/A
**Generality**	Not domain specific	Not domain specific	Not domain specific	Not domain specific	Not domain specific
**Ontology evaluation**	Competency questions	Competency questions and formal axioms	No evaluation method is provided	An informal evaluation method is used for the Chemical ontology	The community evaluates the ontology; agreement process
**Distributed/decentralized**	No	No	No	No	Yes
**Usability**	N/A	Business and foundational ontologies	N/A	Chemical ontology	N/A
**Supporting software**	N/A	N/A	N/A	WebODE	N/A

**Table 2:** Example of the structure of linguistic definitions.

Word	Investigation
**Verb/Noun**	Noun
**Definition**	An Investigation is a set, a collection of related studies and assays; a self-contained contained unit of scientific enquiry.
**Context**	Evaluating the effect of an ingredient in a diet traditionally relies on one or more related studies for example where the subject receive different concentrations of the ingredient. The concept of investigation provides a container that allows us to group these studies together.
**Notes**	When can we consider an investigation completed? Ongoing discussion. For instance, according to the Minimal Information About a Microarray Experiment (MIAME) an Experiment is a set of related hybridization that are in some way related (*e.g*., related to the same publication). In the case of the Investigation, we do not want to tie this concept to a publication or a deposition to a database or a submission to regulatory authority. The decision should be left to the individual investigator.

**Table 3:** Examples of competency questions

Which investigations were done with a high-fat-diet study?
Which study employs microarray in combination with metabolomics technologies?
List those studies in which the fasting phase had as duration one day.
